# Sensitive Adsorptive Voltammetric Method for Determination of Bisphenol A by Gold Nanoparticle/Polyvinylpyrrolidone-Modified Pencil Graphite Electrode

**DOI:** 10.3390/s16060756

**Published:** 2016-05-25

**Authors:** Yesim Tugce Yaman, Serdar Abaci

**Affiliations:** 1 Department of Chemistry, Graduate School of Science and Engineering, Hacettepe University, Ankara 06800, Turkey; tugce.yaman@hacettepe.edu.tr; 2 Department of Chemistry, Analytical Chemistry Division, Hacettepe University, Beytepe, Ankara 06800, Turkey

**Keywords:** Bisphenol A, gold nanoparticle, polyvinylpyrrolidone, pencil graphite electrode, voltammetry

## Abstract

A novel electrochemical sensor gold nanoparticle (AuNP)/polyvinylpyrrolidone (PVP) modified pencil graphite electrode (PGE) was developed for the ultrasensitive determination of Bisphenol A (BPA). The gold nanoparticles were electrodeposited by constant potential electrolysis and PVP was attached by passive adsorption onto the electrode surface. The electrode surfaces were characterized by electrochemical impedance spectroscopy (EIS) and scanning electron microscopy (SEM). The parameters that affected the experimental conditions were researched and optimized. The AuNP/PVP/PGE sensor provided high sensitivity and selectivity for BPA recognition by using square wave adsorptive stripping voltammetry (SWAdSV). Under optimized conditions, the detection limit was found to be 1.0 nM. This new sensor system offered the advantages of simple fabrication which aided the expeditious replication, low cost, fast response, high sensitivity and low background current for BPA. This new sensor system was successfully tested for the detection of the amount of BPA in bottled drinking water with high reliability.

## 1. Introduction

Bisphenol A (BPA) is an important raw material and is extensively used in the packaging industry. It can be used as an antioxidant and inhibitor in manufacturing of polyvinyl chloride (PVC). It can also be used as a monomer for the production of polyacrylat, polycarbonate (PC) and epoxy resin [1]. BPA is a simple hydrocarbon molecule and can bind other molecules such as polystyrene and polycarbonate to form a polymer [2]. In the plastics industry, these materials have a very wide application area such as in reusable plastic bottles, bottles, plates, cups, and containers used in microwave ovens. The epoxy resins are used as linings agent in metal food and beverage cans, glass jars, bottles and metal containers [3]. With the use of products containing BPA, BPA contamination can occur from the package to food and drink. Hydrolysis of the ester bond linking of BPA molecules can accelerate under some conditions such as pasteurization, canning operations and microwave heating before service. These conditions can increase the migration of BPA in consumed products as a result of the heating process. BPA is an endocrine mimic [4], which acts as an estrogen hormone and disrupts the binding mechanism of estrogen-estrogen receptors. It also causes a decrease in sperm quality, and weakness of the immune system, thereby increasing the risk of cancer [2]. Because of the widespread usage of BPA in the packaging industry and the many negative physiological effects, even at low concentrations, it maintains its importance in terms of food security.

In order to determine Bisphenol A, several methods such as chromatographic, spectroscopic [5,6,7,8,9,10,11,12] and electrochemical, have been developed. Although chromatographic and spectroscopic methods are highly sensitive and have low detection limit, they have high costs, a time-consuming process and require trained technicians. This fact has pushed researchers to develop easier and more rapid alternative techniques such as electrochemical methods. Electrochemical characterization and determination of BPA have been carried out in various studies. At bare boron doped diamond and carbon paste electrodes, electron transfer rates were slow and sensitivity was low [13,14]. Thus, the electrode surfaces were modified with different materials to improve the sensitivity [15,16,17,18,19,20,21,22,23].

Pencil graphite electrode (PGE) is a new type of carbon electrode and has widespread use in recent years. PGE has been used for the determination of a wide variety of analytes by various voltammetric techniques [24,25,26,27,28]. PGE has several advantages compared to other carbon-based electrode such as low cost, no need for time-consuming processes like surface polishing and disposability. The surface can be modified easily, has high electrochemical reactivity and surface area. PGE can be also used for stripping voltammetric analysis instead of mercury-based electrodes [27,29,30]. PGE was used for the electrochemical detection of BPA in two studies. Kanatharana *et al.* proposed polyaniline (PANI) nanorods/multi-walled carbon nanotubes (MWCNTs)-modified PGE surfaces for the determination of BPA by amperometry [31]. In another study, Özcan developed electrochemically pretreated PGE with synergistic effect of LiOCl_4_ and NaOH by using adsorptive stripping differential pulse voltammetry [32].

In order to open a new window in the determination of Bisphenol A, it was planned to combine and deposit a nanomaterial like gold nanoparticles (AuNP) and a polymer (polyvinylpyrrolidone) on a pencil graphite surface. AuNP has been used in physics, chemistry, biology, materials science, medical studies and between different disciplines, due to its electronic, optical, thermal and catalytic properties [33]. AuNP causes electrochemical active surface areas to increase [34]. Other important characteristics of AuNP can be considered to be a high surface/volume ratio, high surface energy and the ability to create an electron conduction path between the electrode surface and the analyte [35]. Polyvinylpyrrolidone (PVP) is a water-soluble and long chain polymer which has C=O, C–N and CH_2_ functional groups. It can be used as a surface stabilizer in nanoparticle synthesis. It has some advantages such as nontoxicity, biocompatibility and high surface activity [36]. In addition to these advantages, it provides strong adsorption ability to the analyte for the electrode surface. These properties of PVP are a good indication of ability of this surface as a sensor application.

In this study, an AuNP-PVP-modified PGE surface was prepared for the first time. The purpose was to submit a new, efficient, effortless surface for the determination of trace levels of BPA. It was also intended to indicate that this surface can have much better or comparable analytical performance characteristics with respect to the current literature data for the determination of Bisphenol A. AuNP-PVP-modified PGE surfaces were characterized with electrochemical impedance spectroscopy (EIS) and scanning electron microscopy (SEM). Bisphenol A oxidation was investigated by cyclic voltammetry and it was seen that the oxidation potential of BPA shifted to more positive potential due to the catalytic effect of the AuNP-PVP composite. Parameters that affect the performance of the determination were investigated and optimized. The interference effects of 2,4-Dinitrophenol, p-Nitrophenol, o-Nitrophenol 50-fold concentration; 200-fold concentration of Cd^2+^, Pb^2+^, Hg^2+^, Cu^2+^, and Fe^3+^ were examined. The proposed process was successfully applied to define BPA in bottled drinking water with high reliability. This new sensor system offered the advantages of simple production, rapid response, low cost, high sensitivity and low background current for BPA detection.

## 2. Experiments

### 2.1. Reagent and Apparatus

BPA, HAuClO_4_ and PVP were purchased from Sigma-Aldrich. BPA stock solution (0.1 M) was prepared with absolute ethanol. This stock solution was cold-stored (4 °C) and kept in dark place. Phosphate buffer solution (PBS) was prepared by mixing stock solution of 0.1 M NaH_2_PO_4_ and 0.1 M Na_2_HPO_4_ with redistilled deionized water. HCl or NaOH was used for adjusting the pH.

Interface 1000 Potantiostat/Galvonastat/ZRA was used for all electrochemical experiments with a three-electrode cell system. A disposable pencil graphite electrode was used as a working electrode. The reference electrode was Ag/AgCl and a platinum wire was used as an auxiliary electrode.

### 2.2. Preparation of Pencil Graphite Electrode

The pencil graphite electrode was handmade. It was prepared by using mechanical pencil (Rotring, Germany) as a holder. Pencil lead (diameter of 0.5 mm, HB, Tombow, Tokyo, Japan) was purchased from a local bookstore; 1 cm of lead was modified and immersed in solution per measurement.

### 2.3. Preparation of AuNP/PVP/PGE Surface

Pencil graphite electrodes were located horizontally on the glass surface then 10 μL of PVP solution was dropped through electrode surfaces once and dried for 10 min at room temperature. After this step, 50 μg/mL HAuClO_4_ solution (in 0.5 M H_2_SO_4_) was used for the electrochemical deposition of AuNP on PGE at −0.3 V *vs.* Ag/AgCl by constant potential electrolysis. The electrochemical deposition of AuNP lasted for 60 s.

### 2.4. Analytical Procedure

The electrochemical characterization of bare, AuNP-modified, PVP-modified and AuNP/PVP-modified PGEs were performed by electrochemical impedance spectroscopy. Alternative current (AC) impedance measurements were controlled at the open-circuit value; 0.20 V and the frequency was varied over the range 10^5^–10^–1^ Hz with amplitude of 5 mV in 5 mM K_3_Fe(CN)_6_/K_4_Fe(CN)_6_ containing 0.1 M KCl. The supporting electrolyte (pH 7.0, 0.1 M Phosphate-buffer solution (PBS)) was transferred in to a 5 mL voltammetric cell and was kept in nitrogen atmosphere for 5 min. The square wave adsorptive stripping voltammetry (SWAdSV) method was used for the determination of BPA. The pulse amplitude was of 40 mV, pulse size of 5 mV and frequency of 50 Hz were used for square wave voltammetry. Voltammetric curve was recorded in a potential range from 0.20 to 0.85 V (*vs.* Ag/AgCl). The oxidation peak of Bisphenol A was observed at 0.63 V (*vs.* Ag/AgCl) on AuNP/PVP/PGE.

## 3. Results and Discussion

### 3.1. Characterization of AuNP/PVP-Modified PGE

**Figure 1:**
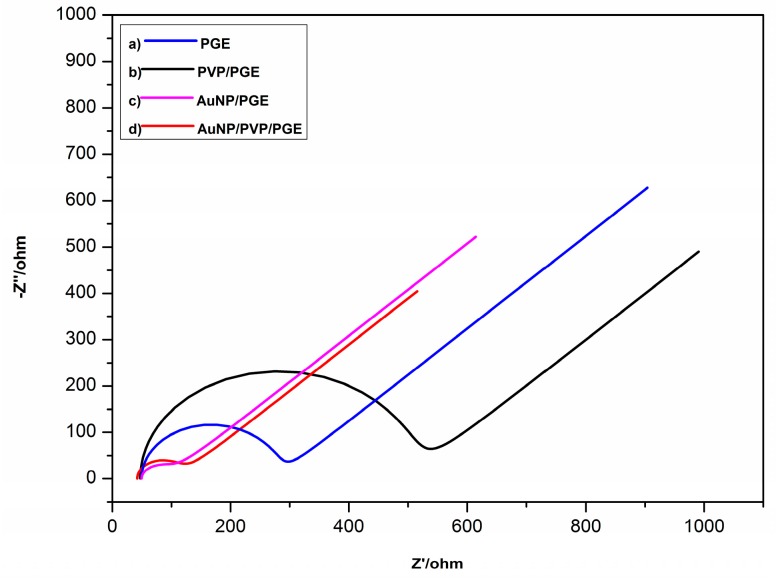
Nyquist diagrams of: (**a**) pencil graphite electrode (PGE); (**b**) polyvinylpyrrolidone (PVP)/PGE; (**c**) gold nanoparticle (AuNP)/PGE; (**d**) AuNP/PVP/PGE in 5 mM Fe(CN)_6_^3−/4−^ 0.1 M KCl (E: 0.2 V; Frequency: 100,000–0.1 Hz).

AuNP/PVP-modified PGE surfaces were investigated using an electrochemical method and scanning electron microscopy. EIS was used as an electrochemical methods (Figure 1).

EIS is a very useful method to get information about properties of the modified surfaces. Figure 1 shows EIS spectrums of bare and modified PGE surfaces. Electron charge transfer resistance (Rct) was calculated for all surfaces by using the Randles equation. The Rct values were found for bare PGE as 227 Ω, PVP-modified PGE as 455 Ω, AuNP-modified PGE as 47 Ω and AuNP/PVP-modified PGE as 71 Ω (relative standard deviation (RSD) < 5%). PVP polymeric film blocked the oxidation and reduction of Fe(CN)_6_^3−/4−^ redox couple by creating resistance between the electrode and the solution interface (Figure 1b). When AuNP was deposited on the surface, resistance was decreased (Figure 1c). This situation can be attributed to the catalytic effect of AuNP. AuNP has electrical conductivity so electrical conductance restriction caused by polymeric film was overcome by the deposition of AuNP on the polymer film (Figure 1d). These results proved that AuNP/PVP-modified PGE surface has a faster electron transfer rate than bare PGE.

**Figure 2:**
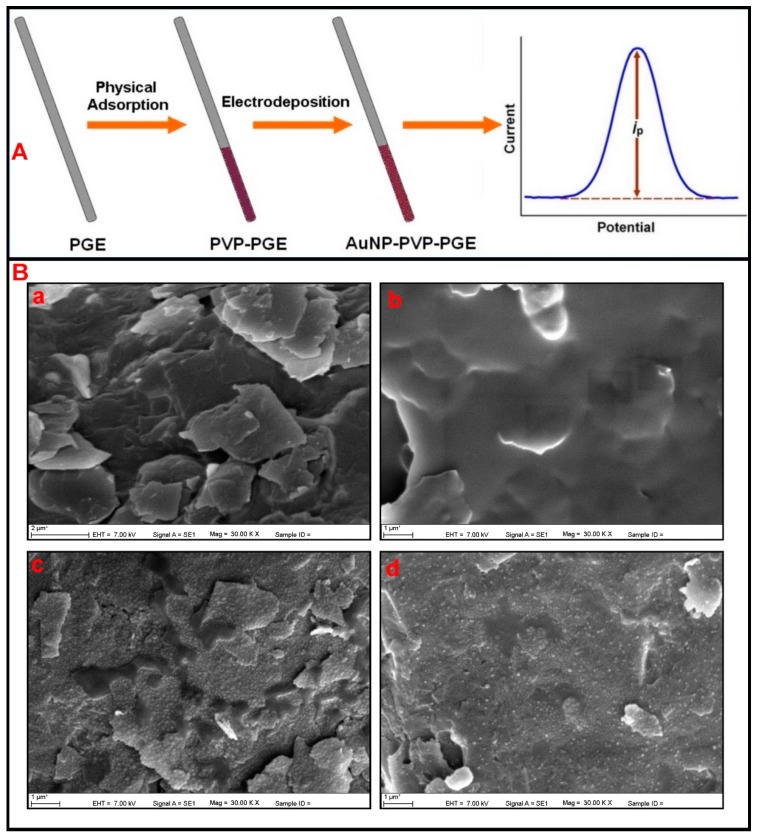
(**A**) Schematic presentation of the work; (**B**) scanning electron microscopy (SEM) images of (**a**) PGE; (**b**) PVP/PGE; (**c**) AuNP/PGE; (**d**) AuNP/PVP/PGE.

SEM was used for the microscopic investigation of electrode surfaces. SEM images of bare and modified PGEs are given in Figure 2B.

Figure 2B(a) shows SEM images of bare PGE. The bare PGE surface has an irregular graphite layer and a rough surface. After the physical adsorption of PVP, irregular graphite layers were covered by polymeric film. This PVP film decreased the roughness of the surface (Figure 2B(b)). AuNP can also be observed in Figure 2B(c,d) with a spherical shape (average radius < 100 nm).

### 3.2. Electrochemical Behavior of Bisphenol A

**Figure 3:**
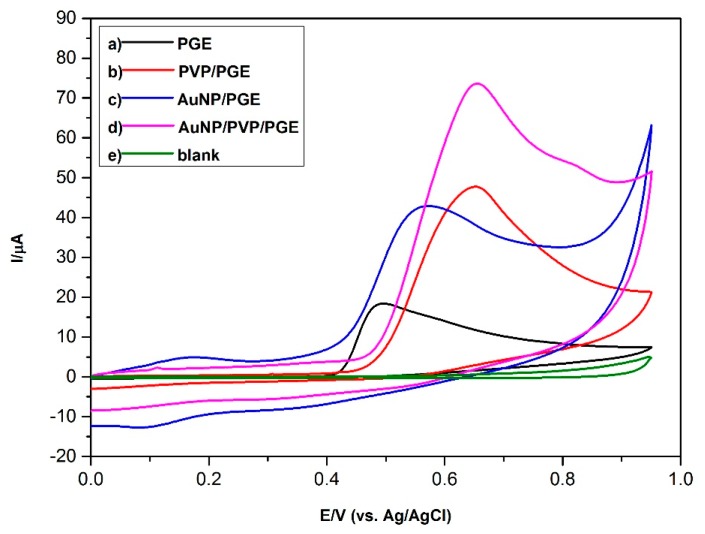
Cyclic voltammograms of 0.1 mM Bisphenol A (BPA) at: (a) bare PGE; (b) PVP-modified PGE; (c) AuNP-modified PGE; (d) AuNP/PVP-modified PGE; (e) absence of BPA at bare PGE. Conditions: pH: 7.0 (0.1 M Phosphate buffer solution (PBS)); scan rate: 100 mV/s^−1^; E_initial_ (Ei): 0.00 V; E_final_ (Ef): 0.95 V.

The electrochemical behavior of 0.1 mM BPA was investigated by cyclic voltammetry at modified and bare electrode surfaces. As can be seen from Figure 3a, the oxidation peak current of BPA was smallest for bare PGE (15.6 μA) with the oxidation potential as 0.49 V *vs.* Ag/AgCl. When the AuNP/PVP/PGE surface was used, the oxidation peak potential of BPA shifted to 0.67 V *vs.* Ag/AgCl, and the peak current increased to 64.0 μA (Figure 3d). This result shows the catalytic effect of AuNP/PVP surface on the BPA oxidation peak current. This effect can be attributed to increased surface area and facilitating electron transfer with the AuNP and strong adsorption ability of BPA with the help of PVP. In summary, it was derived from these results that AuNP/PVP/PGE is suitable for the determination of BPA.

### 3.3. Effect of Scan Rate

**Figure 4:**
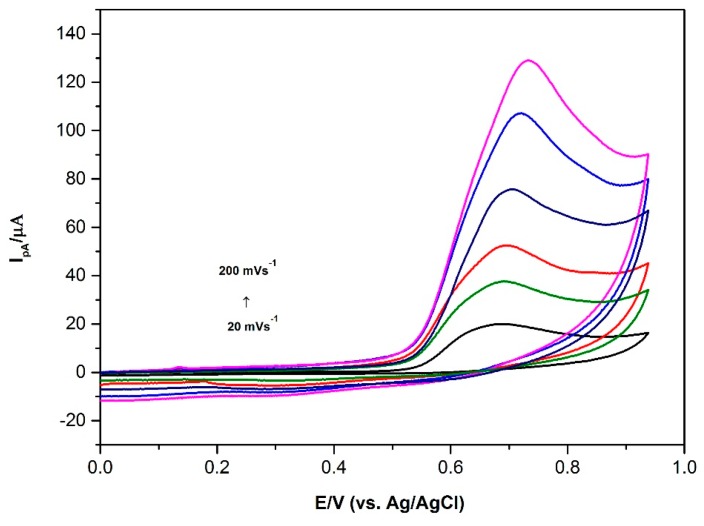
Cyclic voltammograms of 0.1 mM BPA at AuNP/PVP/PGE with different scan rates. Curve bottom to top is obtained at 20, 40, 60, 100, 150, 200 mVs^−1^, respectively. Conditions: pH 7.0 PBS; Ei: 0.00 V; Ef: 0.95 V.

Scan rate study was carried out in a range of 20–200 mVs^−1^ (Figure 4). The solution contained 0.1 mM BPA and has a pH of 7 with PBS. The relation between the anodic peak current and the scan rate can be expressed as follows (Equation (1)),

(1) $$I_{pa}\left(\mu A\right)=0.381\;\vartheta \;\left(mVs^{-1}\right)+7.084\;\left(\;R^{2}=0.998\right)$$

The linearity of this relationship proved that the oxidation peak of BPA on an AuNP/PVP/PGE surface is an adsorption-controlled process. Likewise, the relationship between the $E_{pa}$ and Napierian logarithm of (ln) also had linearity. The obtained equation was (Equation (2)),

(2) $$E_{pa}\left(V\right)=0.025\;\text{ln}\vartheta \;+0.598\;\left(R^{2}=0.999\right)$$

This result also confirmed that BPA oxidation is an adsorption-controlled and totally irreversible process. For this electrode process, peak potential ($E_{pa}$) is given with the formula given below (Equation (3)):

(3) $$E_{pa}=E^{0}+\left(\frac{RT}{\propto nF}\right)\text{ln}\left(\frac{RTk^{0}}{\propto nF}\right)+\left(\frac{RT}{\propto nF}\right)\text{ln}v$$

where *R*, *T* and *F* have usual meaning; *n* is electron transfer number; α is transfer coefficient. The number of electrons transferred during the oxidation of BPA was calculated by using the slope of this equation. In general, α is accepted to be 0.5 in totally irreversible electrode processes [37]. In this study, the slope of $E_{pa}$ *vs.* ln was found to be 0.025. Therefore, the value of α.n was calculated to be 1.032. So, the number of electron (*n*) transferred was calculated as 2.

### 3.4. Optimization of Experimental Parameters

#### 3.4.1. Effect of PVP Concentration and AuNP Deposition Time

**Figure 5:**
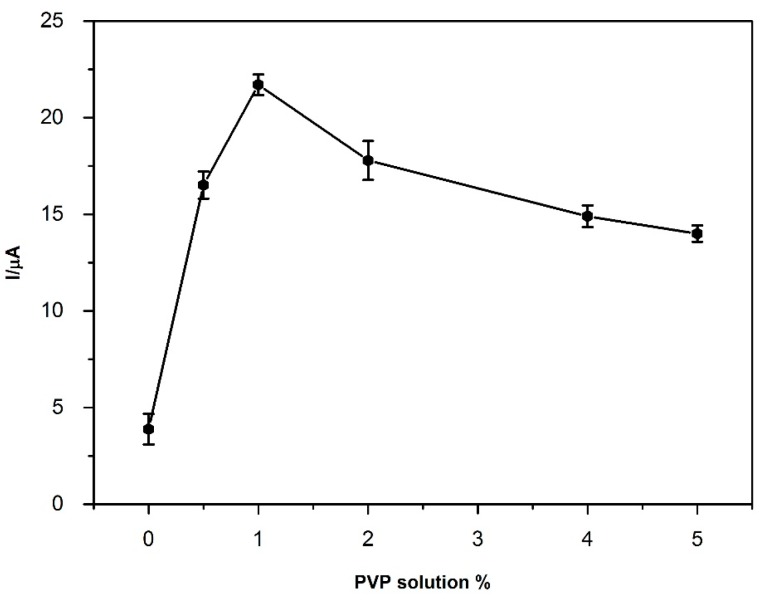
Effect of PVP concentration to peak current of 5 μM BPA. Conditions: pH 7.0; Frequency: 50 Hz; Step amplitude: 40 mV; pulse amplitude: 5 mV; E_deposition_ (E_dep_): −0.2 V; t_deposition_ (t_dep_): 60 s.

PVP (0.5%–5%) solutions with various concentrations were prepared with deionized water and the effect of the PVP concentration on BPA oxidation peak current was examined. The results were shown in Figure 5 and, as can be seen from this figure, the solution with 1% PVP gave the best response. After this point, BPA oxidation peak was decreased considerably. It was determined that when the PVP amount is over 1%, the adsorption capacity of the surface for BPA deposition is too much for the stripping of BPA. Therefore, solution with 1% PVP was chosen as the optimum PVP consideration.

The electrodeposition of AuNP was carried out at −0.3 V (*vs.* Ag/AgCl) for various time intervals by constant potential electrolysis. The oxidation peak current of BPA increased up to 60 s deposition time, but after this value, the peak current of BPA started to decrease. This result showed that deposition times longer than 60 s decreases the electro-catalytic activity of AuNP. Therefore, a 60 s deposition time was found to be the optimum value.

#### 3.4.2. Effect of pH

**Figure 6:**
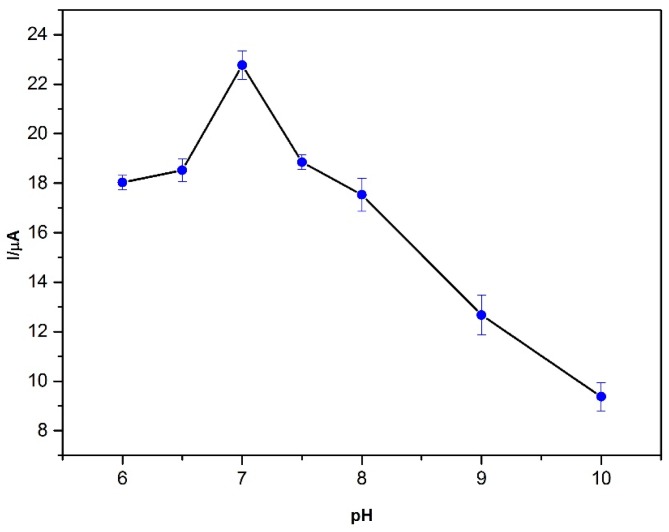
Effects of pH value on the current response of 5 μM BPA. Conditions: pH 7.0; frequency: 50 Hz; step amplitude: 40 mV; pulse amplitude: 5 mV; E_dep_: −0.2 V; t_dep_: 60 s.

The pH study was also carried out as can be seen from Figure 6 by using SWAdSV. It was determined that maximum oxidation peak current of BPA was obtained at pH 7.

**Figure 7:**
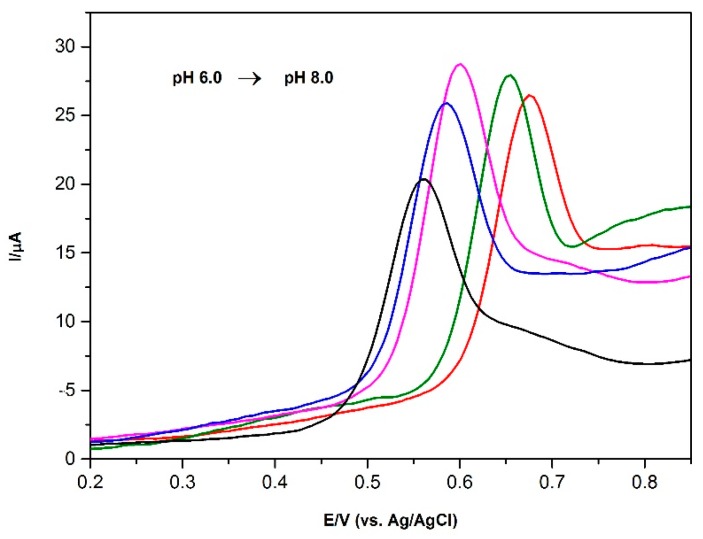
Square wave adsorptive stripping (SWAdS) voltammograms of 2 μM BPA at AuNP/PVP/PGE in different pH (6.0, 6.5, 7.0, 7.5, 8.0) in 0.1 M PBS. Conditions: pH 7.0; frequency: 50 Hz; step amplitude: 40 mV; pulse amplitude: 5 mV; E_dep_: −0.2 V; t_dep_: 60 s.

The relationship between the oxidation peak potential and the pH is shown in Figure 7; a linear shift of Epa towards negative potential with an increasing pH indicates that protons are directly involved in the oxidation of BPA. It obeys the following equation (Equation (4)):

(4) $$E_{pa}\left(V\right)=-0.058\;pH+1.023\;(R^{2}=0.995)$$

A slope of 0.058 VpH^−1^ suggests that the number of electron transfer is equal with that of hydrogen ions taking part in the electrode reaction. A slope of 0.058 VpH^−1^ is approximately close to the theoretical value of 0.0576 VpH^−1^, indicating that the electron transfer is accompanied by an equal number of protons in the electrode reaction [22].

Considering that the number of electrons and protons involved in the oxidation process of BPA is equal to a pH-dependent electrochemical response, the electro-oxidation of BPA at AuNP/PVP/PGE is a two-electron and two-proton process.

#### 3.4.3. Effect of Accumulation Potential and Accumulation Time

**Figure 8:**
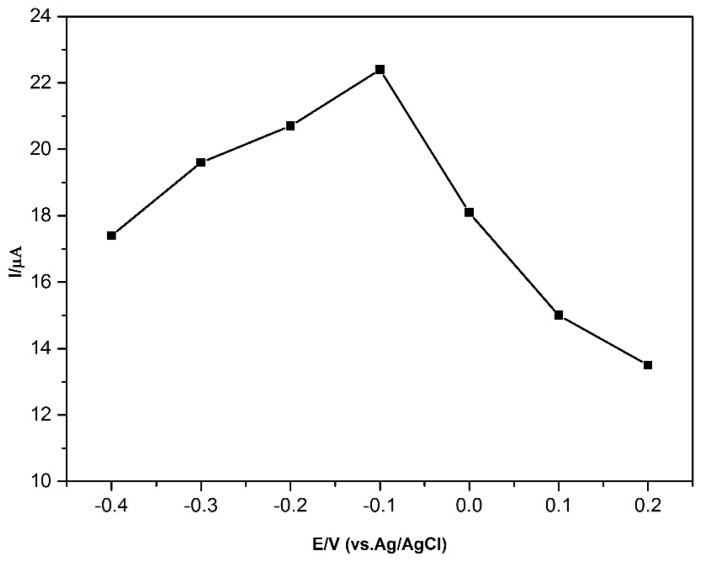
Effect of accumulation potential on the oxidation peak current of 2 μM BPA (t_dep_: 60 s). Conditions: pH 7.0; frequency: 50 Hz; step amplitude: 40 mV; pulse amplitude: 5 mV.

Stripping analysis has two steps, preconcentrating and then stripping. In the preconcentrating step, constant potential was applied for a certain time under stirring. The accumulation time and accumulation potential are too important for the sensitive determination, so these two parameters were investigated and results are shown in Figure 8. The effect of the accumulation potential was examined over the potential range −0.40 to 0.20 V (*vs.* Ag/AgCl) (Figure 8). As shown in Figure 8, the oxidation peak current increased sharply with the changing potential from 0.20 to −0.10 V (*vs.* Ag/AgCl). The oxidation peak current significantly decreased at more negative potential than −0.10 V (*vs*. Ag/AgCl). So, −0.10 V was chosen as the optimum accumulation potential in the following measurements.

The second step was the accumulation time, which was studied in the range of 30 s–210 s with 30 s intervals. The oxidation peak current of BPA increased up to 120 s and then did not change significantly. After this time, this result showed that the modified surface reached the saturation, so the peak current did not increase further. Therefore, 120 s was chosen as the optimum accumulation time in the following measurements.

## 4. Analytical Properties

### 4.1. Linear Range, Limit of Detection and Reproducibility of the Method

**Figure 9:**
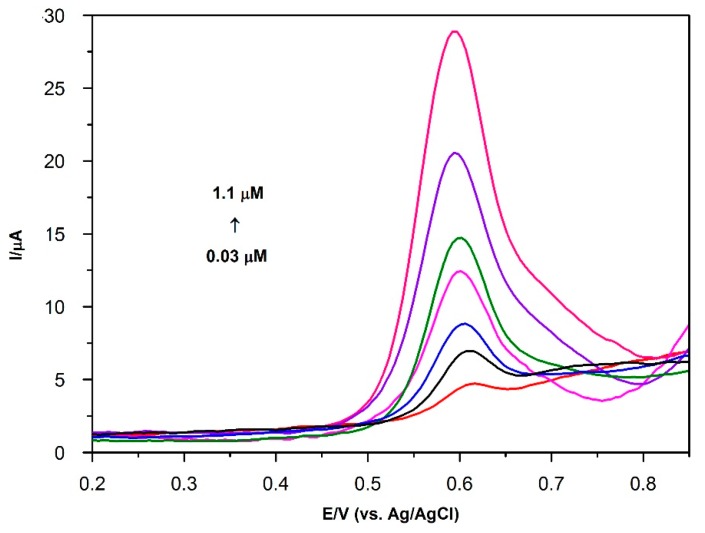
SWAdSV curves of BPA at AuNP/PVP modified PGE in 0.1 M PBS containing 0.03–1.10 μM. Conditions: pH 7.0; frequency: 50 Hz; step amplitude: 40 mV; pulse amplitude: 5 mV; E_dep_: −0.1 V; t_dep_: 120 s.

The calibration curve was obtained for the determination of BPA under optimum experimental conditions. It is given in Figure 9. This curve was derived from the SWAdSV data obtained with AuNP/PVP/PGE surface. The calibration plot of peak current *vs.* concentration was found to be linear over the range of 0.03 μM to 1.1 μM. This linearity is given by the equation given below (Equation (5)):

(5) $$I\left(\mu A\right)=21.71\;C\;\left(\mu M\right)+0.915\;\left(R^{2}=0.999\right)$$

The formula 3 s/slope and 10 s/slope (s is the standard deviation of the blank solution (*n* = 3)) were used for the estimated limit of detection (LOD) and limit of quantity (LOQ), respectively. The LOD and LOQ were found to be 1.0 nM and 3.3 nM, respectively. The reproducibility for the modified electrode was determined by comparing the peak current of 1 μM BPA. The relative standard deviation (RSD) was found to be 1.91% (*n* = 3). It reveals that this fabrication method had good reproducibility.

When these values were compared with the data presented in the literature, it was determined that the developed surface is superior with respect to several studies [31,32,38,39,40,41,42]. Furthermore, AuNP/PVP-modified PGE has good analytical performance from the stand point of low cost, effortless, more reliability and speed. Additionally, our single-used electrode system produced an alternative way to overcome the electrode passivation occurring from the formation of dimers during the oxidation of BPA.

### 4.2. Interference Studies

Interference studies were carried out based on molecules and ions studied in the literature [43,44]. Fifty-fold concentration of 2,4-Dinitrophenol, p-Nitrophenol and o-Nitrophenol had no influence on the signals of BPA with deviations below 5%. Besides some ions such as 200-fold concentrations of Hg^2+^, Pb^2+^, Cd^2+^,Cu^2+^, and Fe^3+^ had no influence on the determination of BPA (RSD 5%). These results showed that this modified electrode has high selectivity for BPA determination.

### 4.3. Analytical Application of AuNP/PVP-Modified PGE

In order to evaluate the analytical performance of AuNP/PVP/PGE in practical applications, the determination of BPA in bottled drinking water samples was performed by using the proposed sensor via a recovery study. The recovery values of BPA were in the range of 99.2% and 103% (Table 1).

These results showed that proposed method was sensitive and effective to determine BPA in bottled drinking water with high reliability.

## 5. Conclusions

A novel AuNP/PVP/PGE-modified electrochemical sensor system was successfully developed for the first time for trace determination of Bisphenol A by using SWAdSV. Parameters affecting the experimental conditions, such as the electrodeposition time of AuNP, concentration of PVP, pH, accumulation time, deposition potential, *etc.*, were investigated for the maximum performance of the electrode. Under optimized conditions, the limit of detection and the limit of quantity were found to be 1.0 nM and 3.3 nM for BPA, respectively, and the voltammetric response showed relatively high sensitivity. The developed surface was shown to be working for the determination of BPA in bottled drinking water with recovery ranging from 99.2% to 103%, which can be a good indication of this surface for applications in real samples with high reliability. To the best of our knowledge, these analytical performance data were better or comparable with respect to the known literature data. In addition, the preparation of the sensor was simple and not a time-consuming process such as enzymatic and labeling, and offered benefits including easy production, low cost, high speed and the highly sensitive detection for BPA. It is our opinion that this new surface has promising features and can be used in other sensor applications.

## Figures and Tables

**Table 1:** Addition and recovery of Bisphenol A from the bottled drinking water by the proposed method at AuNP/PVP/PGE. RSD = relative standard deviation.

Sample	Initial (M)	Added (M)	Found (M)	RSD (%)	Recovery (%)
Bottled drinking water	0.000	5.00 × 10^−8^	(5.10 ± 0.25) × 10^−8^	1.97	102
	0.000	5.00 × 10^−7^	(4.96 ± 0.32) × 10^−7^	2.60	99.2
	0.000	8.00 × 10^−7^	(8.22 ± 0.28) × 10^−7^	1.38	103

Confidence level of 95%, N: 3, t: 4.30.
